# Intraocular Telescopic System Design: Optical and Visual Simulation in a Human Eye Model

**DOI:** 10.1155/2017/6030793

**Published:** 2017-03-30

**Authors:** Georgios Zoulinakis, Teresa Ferrer-Blasco

**Affiliations:** ^1^Department of Optics and Optometry and Visual Sciences, Physics Faculty, University of Valencia, C/ Dr. Moliner 50, 46100 Valencia, Spain; ^2^Interuniversity Laboratory for Research in Vision and Optometry, Mixed Group University of Valencia-University of Murcia, Valencia, Spain

## Abstract

*Purpose.* To design an intraocular telescopic system (ITS) for magnifying retinal image and to simulate its optical and visual performance after implantation in a human eye model. *Methods.* Design and simulation were carried out with a ray-tracing and optical design software. Two different ITS were designed, and their visual performance was simulated using the Liou-Brennan eye model. The difference between the ITS was their lenses' placement in the eye model and their powers. Ray tracing in both centered and decentered situations was carried out for both ITS while visual Strehl ratio (VSOTF) was computed using custom-made MATLAB code. *Results.* The results show that between 0.4 and 0.8 mm of decentration, the VSOTF does not change much either for far or near target distances. The image projection for these decentrations is in the parafoveal zone, and the quality of the image projected is quite similar. *Conclusion.* Both systems display similar quality while they differ in size; therefore, the choice between them would need to take into account specific parameters from the patient's eye. Quality does not change too much between 0.4 and 0.8 mm of decentration for either system which gives flexibility to the clinician to adjust decentration to avoid areas of retinal damage.

## 1. Introduction

Retinal damage results in localized vision loss, and frequently, visual rehabilitation implies optimizing the remaining vision by means of image magnification and/or decentration to nonaffected areas. Age-related macular degeneration (AMD) is a retinal condition that causes a progressive loss of central vision, and its prevalence is being increased due to ageing of the world population and sedentary. Patients diagnosed with AMD face significant and progressive visual loss, which may lead to legal and social blindness [1]. In this situation, patients need to use the peripheral field of view in order to track moving objects and to move in their environment [2–4]. This adds up to the fact that many AMD patients are also afflicted by cataract, where both conditions decrease visual acuity.

Cataract extraction and intraocular lens implantation not only solves satisfactorily the visual decrease caused by the cataract [5] but also improves visual acuity and quality of life in AMD patients, while not influencing the progression of the disease [6]. In this situation, implanting an intraocular telescopic system (ITS) might be an option to consider for optimizing the remaining visual capability of the eye.

An ITS is a miniaturized telescopic device that can be implanted in the human eye. Several trials and research studies have reported the good clinical outcomes, safety, and improved quality of life after implantation [7–10]. These ITS may be grouped into two types: the first one is composed of 2 lenses with high optical power (Galilean telescope) [11] and the second one is composed of mirrors (Cassegrain telescope) [12]. Both ITS project a magnified image, with a magnification of ×2 or ×3, but there is a large variety of different magnifications used in common practice.

Within the Galilean-type ITS, a further division can be made in two more subtypes. The first subtype would be positioned between the anterior and posterior chamber of the eye, while the second one would be positioned completely in the posterior chamber, behind the pupil. The optimal position, distance between the lenses, and magnification provided by these ITS have been reported previously [7, 11, 13].

The purpose of the present study is to design one Galilean telescope of each subtype and simulate optical and visual outcomes in a human eye model to test vision at different vergences. There is also a study and comparison of the quality offered with the decentration of the lenses of each system.

## 2. Materials and Methods

Ray-tracing and optical design software (ZEMAX, USA) was used to design and study the optical and visual quality of the ITS proposed. The human eye model introduced by Liou and Brennan in 1997 [14] was used for the calculations. This eye model is simple enough for the needs of the present study, and while more complicated and recent models could also be used [15–19], the results would follow the same pattern if the model simulates an emmetropic human eye. On the other hand, the main difference between the different theoretical eye models is the way the crystalline lens is designed. For the purposes of this study, as further explained later in the manuscript, the crystalline lens was removed, and therefore, there is no major difference in using one eye model or the other. Unless otherwise stated in the paper, all the parameters of the model used were the same as stated in the original work by Liou and Brennan.

To carry out the simulations, a central incoming field of rays of green light (587.6 nm) passing through a pupil of 3 mm in diameter was used. Two different target vergences were used: a target for distance and a target for near at reading distance (0.41 m). As the target distance decreased, the distance between the lenses had to increase for the image to remain focused.

### 2.1. ITS Design

**Figure 1:**
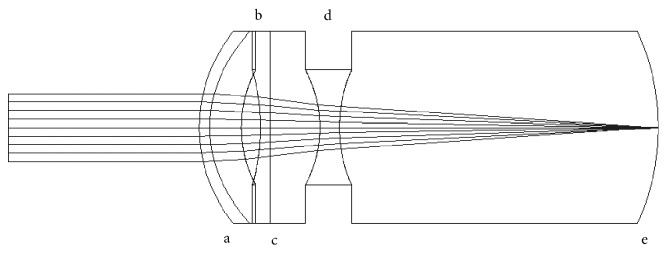
Liou-Brennan eye model with intraocular telescope ITS 1. a, cornea; b, anterior positive lens; c, pupil; d, posterior negative lens; e, retina.

The ITS studied consists of an anterior positive and a posterior negative lens (Galilean telescope). Both lenses were of high optical power as described later in the manuscript. The first ITS designed has the positive lens in front of the pupil and the negative lens behind, while the second one is completely positioned behind the pupil. None of the designs correspond to an existing design, material, or patent. The *ITS through pupil* (ITS 1) was designed following the work of Felipe et al. [11] and the model is shown in Figure 1. The crystalline lens was removed from the eye model, and the empty space was given the refractive index of the aqueous (1.336). The system is composed of a positive anterior lens of 53 diopters (D) and a negative posterior lens of −64 D. The anterior lens was designed and located 1.66 mm from the posterior corneal surface and 0.5 mm in front of the pupil, with a refractive index of 1.55 and thickness of 1 mm. The anterior surface of the lens was given 33 D of power while the posterior surface was calculated to be 20.44 D, to give a total power of 53 D. This power was calculated from the effective power formula

(1) $$\begin{array}{c} D=P_{a}+P_{p}-\frac{t}{n}P_{a}P_{p}. \end{array}$$

In this formula, *D* represents the total optical power in diopters; *P*_*a*_, *P*_*p*_ represent the optical powers of the anterior and posterior surface of the lens, respectively; *t* represents the lens thickness; and *n* represents the refractive index of the lens.

The posterior lens was designed to be located 2.6 mm behind the pupil, with a total power of −64 D. The anterior surface was given a power of −34 D, and the posterior surface was calculated to be −29.36 D. The same thickness and refractive index were used for the power calculation as before. The total distance between both lenses was 3.1 mm for distance and 3.65 mm for near.

**Figure 2:**
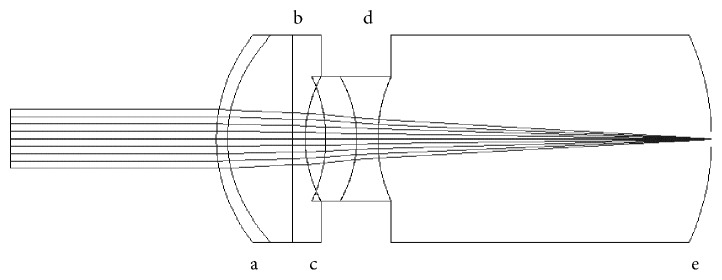
Liou-Brennan eye model with intraocular telescope ITS 2. a, cornea; b, pupil; c, anterior positive lens; d, posterior positive lens; e, retina.

The *ITS behind the pupil* (ITS 2) design was based on the work description of Tabernero et al. [13], and the model is shown in Figure 2. As done previously, the crystalline lens was removed from the eye model. The empty space was given the refractive index of the aqueous, and the whole telescopic system was designed behind the pupil, in the posterior chamber. This system is composed of a positive anterior lens of 66 D and a negative posterior lens of −66 D. For the positive lens, the anterior surface was designed with 36 D and the posterior surface 30.71 D of dioptric power. For the negative lens, the anterior surface was designed with −36 D and the posterior −29.32 D of dioptric power. All calculations were carried out as with the previous design, using the formula for thick lenses. Both lenses had a thickness of 1 mm and a refractive index of 1.55. The distance between the lenses in this system was 1.5 mm for distance and 1.95 mm for near.

#### 2.1.1. Optimization and Decentration of the Lenses

Both ITS were studied under optimized and nonoptimized situations. The optimization process was done using the optimization tool provided by the software. This tool optimizes the system by changing the variables selected by the user in order to get the least root mean square (RMS) wavefront error of the whole optical system. The variables used in this study were the conic constant, the second and fourth asphericity term of the anterior surface of the positive lens of the system. These were selected in order to study the differences between an ITS with spherical lenses and an ITS that also corrects the aberrations produced by the cornea.

**Figure 3:**
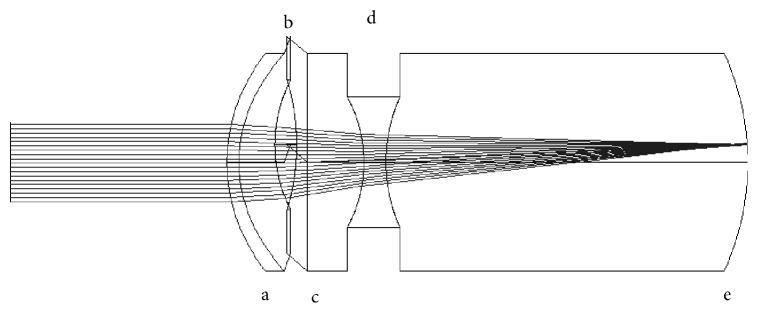
ITS 1 with decentered anterior lens. a, cornea; b, anterior positive lens; c, pupil; d, posterior negative lens; e, retina.

**Figure 4:**
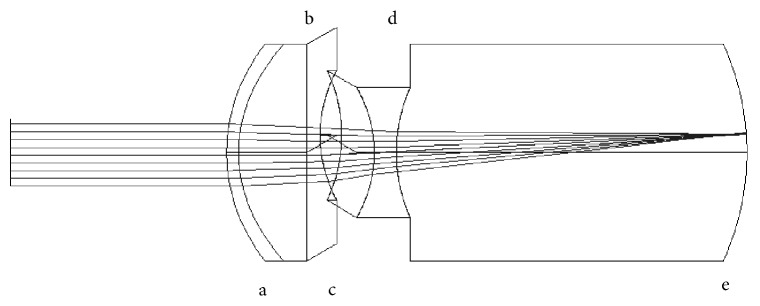
ITS 2 with decentered anterior lens. a, cornea; b, pupil; c, anterior positive lens; d, posterior positive lens; e, retina.

The effect of decentration of the ITS lenses was also explored. In the case of a nonfunctional macula, redirecting the image to a healthy region is one option to be considered. Decentration of the image is provided by a prism effect produced by the decentration of the two lenses. The anterior lens of each ITS was decentered up to 1 mm in 0.2 mm steps. The decentration was done for both optimized and nonoptimized systems. Decentration was induced in one direction only (*y*-axis), since the eye model used is rotationally symmetric. In a customized model (with astigmatism and decentered surfaces), the direction of the decentration would have to be chosen according to the astigmatism and the retinal area where the image needs to be projected on. Figures 3 and 4 show the decentered ITS 1 and 2 designs, respectively.

In order to decenter the lenses, two more surfaces were added on top of the surfaces of each lens. These surfaces are called *coordinate break surfaces*, and they help the user to decenter the lens from the optical axis. They do not alter the final optical and visual quality outcomes in any way, as they only serve as a tool for changing the position of each lens. After performing ray tracing through the optical design software, resulting wavefront RMS error and Zernike coefficients were collected and fed into a custom-made program in MATLAB to calculate a metric called visual Strehl ratio (VSOTF) [20, 21]. The VSOTF is based on the optical transfer function of the whole optical system. It is considered to be one of the best metrics for assessing retinal image quality and has been used in research studies [22, 23]. It is calculated as a ratio of the system's integrated optical transfer function modulated by the contrast sensitivity function to its equivalent for a diffraction-limited system,

(2) $$\begin{array}{c} VSOTF=\frac{\int_{-\infty }^{\infty }\int_{-\infty }^{\infty }{CSF}_{N}\left(f_{x},f_{y}\right)*OTF\left(f_{x},f_{y}\right){df}_{x}{df}_{y}}{\int_{-\infty }^{\infty }\int_{-\infty }^{\infty }{CSF}_{N}\left(f_{x},f_{y}\right)*{OTF}_{DL}\left(f_{x},f_{y}\right){df}_{x}{df}_{y}}, \end{array}$$

where OTF(*f*_*x*_, *f*_*y*_) represents the optical transfer function, OTF_DL_(*f*_*x*_, *f*_*y*_) represents the diffraction-limited optical transfer function, CSF_N_(*f*_*x*_, *f*_*y*_) is the neural contrast sensitivity function, and (*f*_*x*_, *f*_*y*_) are the spatial frequency coordinates [21].

**Figure 5:**
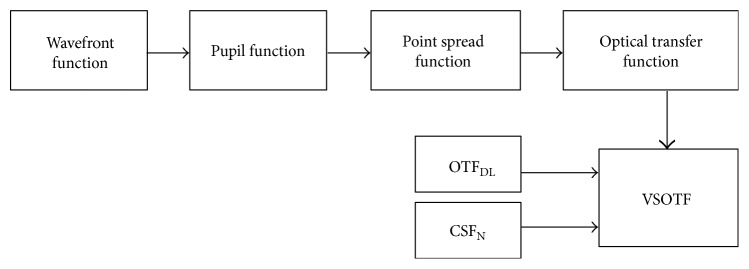
A schematic diagram of the custom algorithm written in MATLAB.

The software program uses the wavefront function, which is produced from the optics of the model eye, the telescope, and the pupil function implemented in the software (circular with 3 mm in diameter). By combining these, it calculates the pupil function that, after Fourier transformation, provides the point-spread function. A secondary Fourier transform yields the optical transfer function (OTF) of the system. The program also calculates the diffraction-limited OTF (OTF_DL_) and the neural contrast sensitivity function (CSF_N_) of the system [24]. Finally, it combines the OTF, OTF_DL_, and CSF_N_ in order to calculate the VSOTF of the system.  Figure 5 provides a graphical approach to the algorithm.

## 3. Results

The optical quality was measured in terms of total wavefront RMS error (for 587.6 nm wavelength) and the visual quality in terms of VSOTF metric. Results for both optimized and nonoptimized telescopic systems were gathered, with either centered or decentered lenses in order to study the impact of decentration in the quality of vision.

Table 1 presents the results for both telescopic systems at far target distance.

**Figure 6:**
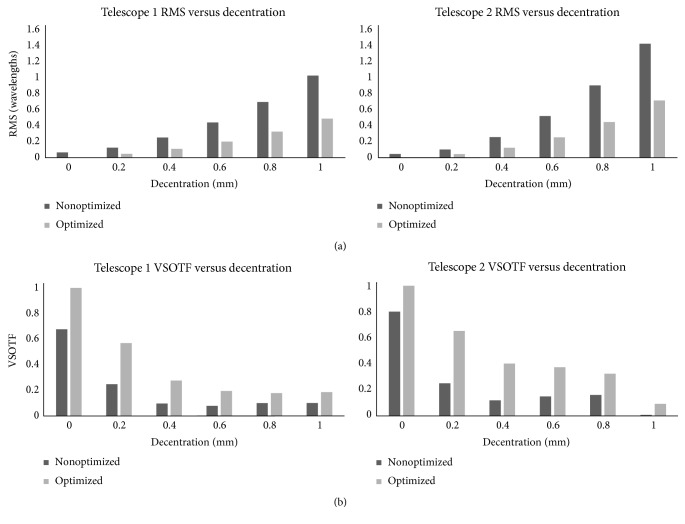
Telescope root mean square (RMS) wavefront error (a) and visual Strehl ratio (VSOTF) (b) versus decentration of the anterior lens.

Figure 6 represents graphically the optical quality results for distance in terms of wavefront RMS error. The wavefront RMS error results were calculated through the ray-tracing software, and they were measured for 587.6 nm wavelength. Figure 6 also shows the visual quality results for distance in terms of the visual Strehl ratio. The VSOTF results were calculated through a pupil of 3 mm diameter.

**Figure 7:**
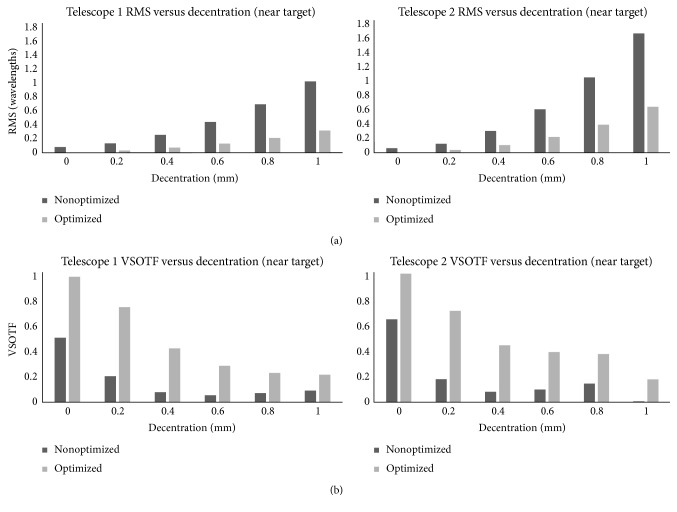
Telescope root mean square (RMS) wavefront error (a) and visual Strehl ratio (VSOTF) (b) versus decentration of the anterior lens for near target distance.

Table 2 presents the results for the first and second telescopic systems at near target distance.  Figure 7 shows graphically the optical and visual results for both telescopic systems focused at near.

When the lenses of each ITS were decentered, the image was also moving towards the peripheral area of the fovea. This image decentration was also measured in the software, and the results for the nonoptimized ITS are shown in Table 3. The same table also compiles the results for the optimized ITS image decentration for distance and near.

## 4. Discussion

Retinal conditions such as AMD have compromised vision in the central field and benefit from magnifying the retinal image or relocating it in order to optimize the remaining visual capabilities. A telescopic system that magnifies and/or projects the image to a healthy part of the retina could be a satisfactory option. In the present study, two different ITS were designed and compared. The first one is composed of an anterior lens of +53 D optical power, positioned in front of the pupil, and a posterior lens of −64 D optical power, placed behind the pupil. The second telescope is totally positioned behind the pupil and is composed of an anterior lens with optical power +66 D and a posterior lens of −66 D. In order to focus at different distances, the distance between the lenses must change as well in both ITS proposed. For the ITS 1, when focused at distance, the distance between lenses was 3.1 mm, increasing to 3.65 mm for near targets. For ITS 2, the distance between lenses changed from 1.5 mm when focused at distance to 1.95 mm when focused at near.

For both designs (see Figure 6), the optical and visual quality is better when using aspheric lenses in order to correct the aberrations induced by the cornea and the implantation procedure. ITS 2 provides better optical and visual results than ITS 1. The same observations can be done from Figures 4 and 5 for the near target results. Both ITS could provide equal quality of vision in AMD patients. The ITS 2 provides slightly better results, and the fact that the whole ITS is behind the pupil and is smaller in length suggests it could be a better option for a real implant.

Another parameter that plays a significant role in the choice of an ITS would be the axial length of the eye. As Felipe et al. [11] stated in their study, longer eyes (myopic) would be more suitable for the ITS 1.

A further expansion of this study could be considered in order to optimize the asphericities of the anterior lens after the decentration of the lens. This could result in better optical and visual quality as previously done in the study by Tabernero et al. [13]. In this study, the optimization was done before the decentration of the lenses in order to test the image quality when the decentration of an already manufactured ITS needs to be selected.

For the near targets, the results follow the same trend with that for distance (Figure 7). The VSOTF decreases as decentration increases. Nevertheless, between 0.4 and 0.8 mm decentration, the difference between the results is minimal. This could indicate a range of selectable decentrations that would allow the clinician to relocate the retinal image without modifying significantly its quality.

In general, while the decentration increases, the quality decreases dramatically. There are astigmatic and coma aberrations induced because of the decentration of the lenses. As Tabernero et al. [13] proposed in their study, a cylinder lens could be used in order to fix the induced amount of astigmatism. On the other hand, as previously discussed, image quality is not significantly affected by decentrations between 0.4 and 0.8 mm for either distance or near targets (Table 3). This decentration induces a displacement of the retinal image within the central 3.5 degrees of the retina, which is within the foveal and parafoveal area.

According to these results, depending on the area of the retinal damage, the surgeon might choose a specific decentration for each patient without altering significantly the quality of the image. For ITS 2 particularly, the calculated VSOTF is above the 0.3 limit that represents the 0 logMAR, as proposed by Cheng et al. [25]. Obviously, as departing from the fovea, the image would be displaced to a retinal region with lower visual capabilities, and therefore, the final visual result might be even lower, but the optical quality provided by the ITS would be better than the visual threshold for that part of the retina.

Long-term results of recent studies [26] report visual results in agreement with our simulations. In the same study, it is also reported that younger patients showed even better results than the older ones, something that is expected as their vision is generally better. These young subjects also presented less adverse events from the application of such devices. In this way, the simulation of these telescopic systems could provide better results in terms of agreement with clinical studies and increase our knowledge in this research field.

In the end, biometric parameters must be determined before considering which of the designs should be considered to be used. Both systems can be used, but there is still space for more research in their designs and applications.

## Figures and Tables

**Table 1:** Optical and visual results for both telescopic systems (far target distance).

	Decentration (mm)	Optimized system		Nonoptimized system	
		RMS (wavelengths)	VSOTF	RMS (wavelengths)	VSOTF
ITS 1	0.0	0.00017	0.99997	0.06621	0.67655
	0.2	0.04750	0.56845	0.12529	0.24763
	0.4	0.11107	0.27600	0.25315	0.09752
	0.6	0.20162	0.19473	0.44053	0.07813
	0.8	0.32587	0.17874	0.69565	0.10008
	1.0	0.48908	0.18612	1.02587	0.10115

ITS 2	0.0	0.00032	0.99997	0.04573	0.80058
	0.2	0.04446	0.65255	0.10011	0.25081
	0.4	0.12208	0.40203	0.25297	0.11908
	0.6	0.24940	0.37397	0.51092	0.14969
	0.8	0.43805	0.32400	0.88700	0.16121
	1.0	0.70249	0.09259	1.40018	0.00757

**Table 2:** Optical and visual results for both telescopic systems (near target distance).

	Decentration (mm)	Optimized system		Nonoptimized system	
		RMS (wavelengths)	VSOTF	RMS (wavelengths)	VSOTF
ITS 1	0.0	0.00050	0.99997	0.08162	0.51337
	0.2	0.03162	0.75714	0.13343	0.20787
	0.4	0.07285	0.42921	0.25653	0.07948
	0.6	0.13094	0.29042	0.44222	0.05522
	0.8	0.21107	0.23356	0.69713	0.07219
	1.0	0.31791	0.21931	1.02795	0.09214

ITS 2	0.0	0.00058	0.99997	0.06363	0.64374
	0.2	0.03885	0.71124	0.12556	0.17792
	0.4	0.10720	0.44347	0.30485	0.07998
	0.6	0.22132	0.39109	0.60986	0.09826
	0.8	0.39469	0.37503	1.05769	0.14287
	1.0	0.64508	0.17847	1.67465	0.00579

**Table 3:** Image decentration for both optimized and nonoptimized ITS over anterior lens decentration.

	Anterior lens decentration (mm)	ITS 1 (nonoptimized)		ITS 2 (nonoptimized)	
		Image decentration far target distance (mm)	Image decentration near target distance (mm)	Image decentration far target distance (mm)	Image decentration near target distance (mm)
Nonoptimized systems	0.2	0.1953	0.1991	0.2082	0.2152
	0.4	0.4016	0.3992	0.4222	0.4240
	0.6	0.6010	0.6017	0.6278	0.6440
	0.8	0.7952	0.7992	0.8346	0.8585
	1.0	1.0130	1.0020	1.0610	1.0810

Optimized systems	0.2	0.1953	0.2041	0.2110	0.2179
	0.4	0.4037	0.3992	0.4227	0.4324
	0.6	0.6051	0.6042	0.6304	0.6440
	0.8	0.7964	0.8042	0.8612	0.8585
	1.0	0.9876	1.0020	1.0780	1.0880
